# Assessment of the Impact of Fruit Vinegars on the Tenderness and Quality Attributes of Spent Hen Meat

**DOI:** 10.1002/fsn3.70544

**Published:** 2025-07-01

**Authors:** Nuran Erdem

**Affiliations:** ^1^ Department of Food Processing, Güzelyurt Vocational School Aksaray University Aksaray Turkey

**Keywords:** chicken, marination, microbial quality, pineapple, scanning electron microscopy, texture, tough meat

## Abstract

This study aimed to evaluate the impact of marinades formulated with fruit vinegars (grapefruit, jujube, pineapple, strawberry) on the physicochemical, structural (Texture Profile Analysis‐TPA, Warner–Bratzler shear force‐WBSF, shear energy‐WBSE, Scanning Electron Microscope‐SEM), microbiological (total mesophilic aerobic bacteria, total psychrotrophic aerobic bacteria, coliform bacteria, *Escherichia coli* , yeast, and mold), and sensory characteristics (odor, color, flavor, texture, overall acceptance) of sample from 83‐week‐old spent laying hens, which are typically known for their tough texture. The samples were manually mixed to evenly distribute the solid components within the marinades, then refrigerated at 4°C for 24 h. Following marination, they were cooked in a convection oven at 160°C for 30 min. The addition of vinegar contributed to lower pH levels and reduced cooking loss in the meat. The highest moisture content and water‐holding capacity were observed in the Cp group. Marination with vinegar caused a significant reduction in L* values, while it resulted in an increase in a* values. SEM images revealed that marination with vinegar led to extensive degradation of connective tissues. Meat marinated with pineapple and strawberry vinegar showed significantly lower hardness, WBSF, and WBSE (*p* < 0.05). These results were further supported by sensory analysis, as texture scores aligned with the TPA and SEM findings. Marination with vinegars caused a significant reduction in microbial counts. The findings indicate that among the vinegars studied, pineapple and strawberry vinegar emerge as effective natural tenderizing agents, enhancing the textural properties of tough meats while potentially improving other quality attributes.

## Graphical abstract

Vinegar addition lowered pH and cooking loss, while the Cp group had the highest moisture and WHC. SEM images revealed that marination with vinegar led to extensive degradation of connective tissues. Meat marinated with pineapple and strawberry vinegar showed significantly lower hardness, WBSF, and WBSE. These results were further supported by sensory analysis, as texture scores aligned with the TPA and SEM findings. Marination with vinegars caused a significant reduction in microbial counts.

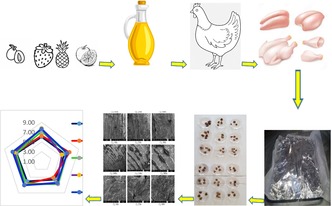

## 1 Introduction

Texture is a critical determinant of meat quality, directly influencing consumer satisfaction, and purchasing decisions. Research by Martinez et al. (2023) underscores the strong relationship between perceived tenderness and juiciness and overall consumer preference. However, meat from older animals often exhibits reduced tenderness due to increased connective tissue cross‐linking. As animals age, mature cross‐links such as pyridinoline accumulate, leading to decreased collagen solubility and a firmer, less desirable texture (Li et al. 2021). Consequently, the meat industry faces challenges in delivering products with consistent quality attributes, including tenderness, juiciness, and flavor. Enhancing meat tenderness is a primary focus of research efforts conducted by scientists worldwide. One potential method for improving meat tenderness is marination. Marination is a common method in meat processing that incorporates ingredients like oil, salt, phosphates, organic acids, sugar, herbs, spices, and aromatic compounds to improve tenderness, moisture retention, and flavor. It also plays a key role in enhancing microbial safety and overall meat quality (Sengun et al. 2021; Somek 2018).

Hens that are no longer capable of laying eggs efficiently are referred to as “spent hens”. Under commercial production systems, laying hens are typically culled between 72 and 95 weeks of age, depending on their productivity and market egg prices. Meat from spent hens is typically characterized by its toughness, lack of juiciness, and low fat content. As hens get older, cross‐linkages develop within their collagen network, resulting in meat that is less juicy and tougher (Aviagen 2021; Kumar et al. 2021; Petek and Çavuşoğlu 2021; Polizer Rocha et al. 2019). The growing population of spent hens presents significant challenges regarding their disposal. Their carcasses are often difficult to market and, in some cases, are discarded. The costs associated with disposing of slaughtered hens and the amount of biological waste generated have presented economic challenges for the poultry industry, while also contributing to significant environmental pollution. Reducing edible waste is a key priority in industrialized nations. Therefore, it is essential to explore efficient alternatives for the utilization of spent hens (Kumar et al. 2021; Petek and Çavuşoğlu 2021).

Vinegar is produced through the fermentation of grains, fruits, sugars, or molasses, including rice, corn, pineapples, apples, oranges, strawberries, mangoes, and Bengal currant. Fermented vinegar is typically clear, with only natural sediment, and possesses a pleasant aroma that reflects its raw materials, along with a well‐balanced taste (Boondaeng et al. 2022). It is commonly incorporated into meals and salads and plays a key role in the preparation and preservation of various food products, including pickles, mayonnaise, tomato paste, and mustard (Sengun et al. 2021). Furthermore, vinegar serves as a valuable source of bioactive compounds, including organic acids (primarily acetic acid, along with tartaric, formic, lactic, citric, and malic acids), phenolic compounds, minerals, vitamins, and melanoidins. Its broad application in food technology is attributed to its ability to combat microbes, prevent oxidation, reduce carcinogenic risks, and fight infections (Sengun et al. 2021; Bakir et al. 2017).

Spent hen meat is underutilized in the food supply chain due to its tough texture, caused by age‐related increases in collagen and connective tissue cross‐linking. This limits consumer acceptance and its use in traditional meat products. Recent research emphasizes the need for processing strategies to improve its palatability and marketability (Lee and Kim 2021). Globally, especially in developing and underdeveloped nations, a significant portion of the population experiences nutritional deficiencies due to limited access to animal‐based protein sources (Sarkar et al. 2020). As a rich source of protein, spent hen meat offers promising potential as a solution to this issue. Acidic marinades enhance meat tenderness by reducing its pH, which causes the muscle structure to weaken, promotes proteolysis through cathepsins, and boosts the conversion of collagen into gelatin (Sengun et al. 2021). For these reasons, incorporating vinegar into marinade recipes is essential. This study aimed to examine the effects of marinating spent hen meat with grapefruit, jujube, pineapple, and strawberry vinegars, followed by convection oven cooking, on its tenderness and physicochemical, structural, microbiological, and sensory characteristics.

## 2 Materials and Methods

### 2.1 Materials

Spent hens (Lohman Brown breed) were obtained from Aksaray, Turkey. Fifteen laying hens, aged 83 weeks and no longer productive, were procured for slaughter. The chicken carcasses arrived at the laboratory within 35 min. Breast meat from each was carefully removed and kept at 4°C for a day. Since this study did not involve any experimental procedures on live animals or birds (spent hens), approval from the Institutional Animal Ethics Committee (IAEC) was not necessary.

Cold‐pressed extra virgin olive oil, rosemary, black pepper, and salt were obtained from local producers and markets in Mersin, Türkiye. Fruit vinegars (grapefruit, jujube, pineapple, and strawberry) were obtained from local producers in Mersin, Türkiye. According to the producers, the vinegars were traditionally prepared through natural two‐step fermentation of fruit juice: first, alcoholic fermentation by *Saccharomyces cerevisiae* , followed by acetic acid fermentation using acetic acid bacteria (mainly *Acetobacter* spp.) under aerobic conditions.

### 2.2 Preparation of Sample

In this research, four marinades were formulated using a range of vinegars: grapefruit, jujube, pineapple, and strawberry. The vinegar concentration in the marinade was adjusted to 30%. The marinades were prepared using the following ingredients: 246 mL of water, 120 mL of vinegar (grapefruit, jujube, pineapple, or strawberry), 30 mL of olive oil, 0.4 g of rosemary, 0.4 g of black pepper, and 3.2 g of salt. A control meat sample was made using water without the addition of any vinegar. In the marinade formulations containing vinegar, 120 mL of water was replaced with 120 mL of the respective vinegar. The sample groups were classified as follows: Cc (control group with no vinegar), Cg (meats marinated with marinades prepared with grapefruit vinegar), Cj (meats marinated with marinades prepared with jujube vinegar), Cp (meats marinated with marinades prepared with pineapple vinegar), and Cs (meats marinated with marinades prepared with strawberry vinegar). The marinades were prepared by mixing all the ingredients for 10 min. The marinades were left at ambient temperature for a minimum of 2 h while being continuously stirred to allow proper hydration of the dried components.

### 2.3 Sample Preparation: Marinade Application and Cooking

Each chicken breast (one piece) meat was cut into rectangular pieces measuring 5 × 3 × 1 cm, with each batch weighing 300 g. Each meat sample was randomly placed in a plastic bag and marinated at a 1:1 meat‐to‐marinade ratio, then refrigerated at 4°C for 24 h. Following the marination, the meats were removed from the marinades. Afterwards, the chicken meat was wrapped in aluminum foil. The spent hen meats were cooked in a convection oven (Nüve‐FN120, Türkiye) at 160°C for 30 min until they reached an internal temperature of 76°C, monitored with a Digital Bratengabel‐TCM thermometer (Tchibo GmbH, Germany).

### 2.4 Analyses in Vinegars

#### 2.4.1 Determination of Acidity, °Brix Content, pH, and Color in Vinegar Samples

The total acidity (% acetic acid) of the vinegars was determined through titration. The°Brix values were obtained with a refractometer (Master‐M, ATAGO, Tokyo, Japan), calibrated using distilled water. The pH levels of the vinegars were measured using a WTW Series pH 720 pH meter in accordance with the methodology described by AOAC (2007). Color measurements were performed using a Chromameter CR‐400 (Konica Minolta, Osaka, Japan) equipped with a D65 illuminant and a 2° standard observer. The device was calibrated prior to each measurement session using a standard white calibration plate (L* = 91.78, a* = −0.43, b* = 3.47). Measurements were taken from the surface of the vinegar samples at three different locations to account for surface variability, and the average of three independent replicates was recorded for each sample. The CIE L*, a*, and b* parameters were used to evaluate lightness, redness/greenness, and yellowness/blueness, respectively, in accordance with standard procedures described in Commission Internationale de l'Éclairage guidelines.

### 2.5 Analyses in Spent Hen Meats

In this study, spent hen meat was marinated with grapefruit, jujube, pineapple, and strawberry vinegars, then cooked in a convection oven, followed by various analyses.

#### 2.5.1 Water Content

The moisture content was assessed following standard procedures outlined by the AOAC (2000).

#### 2.5.2 pH Measurement

The pH of the spent hen meats was measured using a WTW Series pH 720 m, following the guidelines outlined by AOAC (2000). The pH meter was calibrated using standard buffer solutions with pH values of 4.0, 7.0, and 10.0 before performing the measurements.

#### 2.5.3 Color Properties

A Chromameter CR‐400 (Konica Minolta, Osaka, Japan) with illuminant D65 and a 2° observer was used for color determination. The CIE L*, a*, and b* color coordinates were recorded from the visible surfaces of the spent hen meats. The Chromameter was calibrated with a white plate (L* = 91.78, a* = −0.43, b* = 3.47) before each analysis. Color was assessed by taking measurements at five distinct points on the surface of each sample, ensuring a perpendicular angle for accuracy.

#### 2.5.4 Cooking Loss

Cooking loss (CL) was evaluated by quantifying mass variations throughout the marination and cooking stages, following the procedure outlined by Young and Buhr (2000). Sample weights were measured prior to cooking as the marinated raw weight (w1) and after t as follows:

$$\text{Cooking loss}\;\left(\%\right)=\left[\;\left(\text{w1}-w2\right)/\text{w1}\right]\times 100$$

#### 2.5.5 Water‐Holding Capacity (WHC)

An 8 g portion of spent hen meat was combined with 12 mL of a 0.6 M sodium chloride solution (Merck KGaA, Darmstadt, Germany) and placed in a water bath maintained at 5°C ± 1°C for 15 min. The samples were then centrifuged (Nuve, NF‐800‐R Model, Turkiye) at 10,000 rpm for 15 min at 4°C. The resulting supernatant was collected for the determination of the WHC (%) (Wardlaw et al. 1973).

#### 2.5.6 Texture Measurement

The texture profile analysis (TPA) of spent hen meats (21°C) was performed using a TX‐XT Plus Texture Analyzer, employing the two‐fold compression method (Herrero et al. 2007). From each group, five slices were selected for analysis. Each slice underwent two consecutive compressions with a 0.1‐s interval between cycles. The device settings were as follows: a pre‐test speed of 1 mm/s, a test speed of 5 mm/s, a post‐test speed of 5 mm/s, and a compression depth of 50%. The textural properties assessed included hardness (in Newtons), springiness, cohesiveness, gumminess, chewiness (in Newtons), and resilience.

To measure the Warner–Bratzler shear force (WBSF) and shear energy (WBSE), a shear test was conducted using a texture analyzer (TX‐XT Plus) equipped with a V‐notch blade attachment.

#### 2.5.7 Microstructure

Observations and photomicrography of the microstructure of the marinated spent hen meat were conducted using a scanning electron microscope (SEM, FEI, Quanta 650), as described by Mazaheri Kalahrodi et al. (2021). Breast spent hen meats were placed in the SEM, and the meat surface was analyzed at magnifications of 1000× and 5000×.

#### 2.5.8 Microbiological Analyses

The unmarinated raw meat, marinated raw meat, and cooked breast meat were aseptically sampled from various sections of the breast. Total mesophilic aerobic bacteria (TMAB) and total psychrotrophic aerobic bacteria (TPAB) were enumerated using Plate Count Agar (PCA, Merck). TMAB counts were determined by incubating plates at 30°C for 24–48 h, while TPAB were assessed after incubation at 7°C for 8–10 days. *Escherichia coli* and coliform bacteria counts were quantified on Violet Red Bile Agar (VRB, Merck), with samples grown at 37°C for 24 h. Similarly, yeast and mold populations were quantified on Potato Dextrose Agar enriched with 10% lactic acid (PDA, Merck), following a 5‐day incubation at 25°C (Gökalp et al. 1999). Microbiological counts were expressed as log CFU/g, with a detection threshold of 1.0 log CFU/g. Each analysis was performed in triplicate for every sample.

#### 2.5.9 Sensory Evaluation

A sensory assessment was conducted with 60 participants from the Department of Food Processing at Aksaray University, comprising 61% women and 39% men, with ages ranging from 21 to 49. Spent hen meat samples were allocated unique identification codes and presented to participants in a randomized sequence. Water and bread were available as palate refreshers. The panel attended four sessions, each featuring five pieces of breast meat served at 21°C. Each sample was evaluated for odor, color, texture, flavor, and overall acceptance, with ratings ranging from 1 (extremely bad) to 9 (extremely good).

#### 2.5.10 Statistical Analysis

The statistical analyses were performed using Minitab 16.0. ANOVA was conducted, and Tukey's multiple comparison test was applied to determine significant differences among group means at a significance level of *p* < 0.05. All measurements were conducted in triplicate, and the entire experiment was replicated twice.

## 3 Results and Discussion

### 3.1 pH and Acidity, Total Soluble Solid, and Color of Vinegars

The pH, total titratable acidity (g/% acetic acid), °Brix content, and color of the vinegars are presented in Table 1. As shown in Table 1, the pH of the vinegars ranged from 2.98 to 3.58. Bakir et al. (2017) similarly found that the pH levels of vinegars ranged from 2.80 to 3.90. In a separate study, Kang et al. (2020) recorded pH ranging from 2.59 to 2.98 for vinegar samples. The total acidity of the vinegars varied between 0.83% and 1.58%, with pineapple vinegar exhibiting the highest acidity at 1.58% (*p* < 0.05). Similarly, Öztürk (2022) reported that the total acidity of fruit vinegars ranged from 0.53 to 3.23 g of acetic acid per 100 mL. In this study, the°Brix of the vinegars ranged from 1.25 to 2.15. Jujube vinegar had the lowest°Brix value, whereas grapefruit, pineapple, and strawberry vinegars displayed similar levels with no notable variation (*p* > 0.05). Previous studies have reported°Brix values for fruit vinegars ranging between 1.20 and 6.63 °Brix (Bayram et al. 2020; Kang et al. 2020). The L* parameters of the vinegars ranged from 42.69 to 48.90. The a* parameters of jujube and strawberry vinegars were higher than those of grapefruit and pineapple vinegars (*p* < 0.05). Additionally, the b* value of jujube vinegar surpassed that of all vinegars (*p* < 0.05). Bayram et al. (2018) obtained that the L* parameters of vinegars (rice, apple, and grape) ranged from 7.31 to 14.31, the a* values from 0.50 to 12.79, and the b* values from 12.54 to 22.85. The differences observed in similar studies on this topic can be attributed to variations in the raw materials used, production methods, fermentation conditions, and maturation periods.

**TABLE 1:** pH, total acidity, °Brix, and color values of vinegars.

Samples	pH	Total acidity^1^	°Brix	L*	a*	b*
Distilled water	6.75 ± 0.03^a^	—	—	—	—	—
Grapefruit vinegar	3.27 ± 0.02^c^	1.40 ± 0.01^b^	1.95 ± 0.05^a^	48.00 ± 0.32^ab^	0.58 ± 0.10^b^	4.50 ± 0.34^b^
Jujube vinegar	3.58 ± 0.03^b^	0.83 ± 0.02^c^	1.25 ± 0.05^b^	45.20 ± 0.09^bc^	1.16 ± 0.09^a^	7.33 ± 0.32^a^
Pineapple vinegar	2.98 ± 0.04^e^	1.58 ± 0.03^a^	1.95 ± 0.05^a^	42.69 ± 0.11^c^	0.23 ± 0.11^c^	−0.62 ± 0.16^c^
Strawberry vinegar	3.14 ± 0.03^d^	1.49 ± 0.02^ab^	2.15 ± 0.05^a^	48.90 ± 0.06^a^	1.27 ± 0.18^a^	3.43 ± 0.02^b^

*Note:* Mean ± SE. Values in the same column with different superscripts are significantly different (*p* < 0.05). *n* = 3; each value represents the mean of three replicates.

^1^ g acetic acid/100 mL.

### 3.2 pH Values of Spent Hen Meat

The most significant effects of marinade solutions include the weakening of the protein structure, enhanced proteolysis—particularly myosin degradation—and the activation of lysosomal enzymes, which occurs as the environmental pH approaches its optimal activity range (Bhat et al. 2018). Different marinade types significantly influenced the pH of the meats. The pH values among the groups ranged from 4.56 to 6.06, as shown in Table 2. All marinated spent hen meats had a lower pH (*p* < 0.05) compared to the Cc group. The highest pH was determined in the Cc group, whereas the Cp group had the lowest pH measurement. This observation may be attributed to the low pH of pineapple vinegar. The results also indicate that the pH of the marinated sample decreased in line with the lower pH of the fruit vinegars (*p* < 0.05). In a study where meat samples were treated with apple cider vinegar, pomegranate and lemon juices under different temperature–time conditions, it was observed that the pH of chicken meats marinated with lemon juice was lower than that of the other treatments (Lytou et al. 2017). This outcome may be attributed to limited glycogen reserves, a high initial meat pH, and the intrinsic pH and composition of the marinade, particularly the presence of organic acids like acetic acid in vinegar, which can lower the pH of marinated samples (Kim et al. 2013; Mazaheri Kalahrodi et al. 2021).

**TABLE 2:** The moisture content, pH, cooking loss (CL), water holding capacity (WHC) and color (L*, a*, b*) values of spent chicken meats.

Samples	Moisture (%)	pH	CL (%)	WHC (%)	L*	a*	b*
Cc (Control)	61.04 ± 0.45^b^	6.06 ± 0.01^a^	49.65 ± 0.21^a^	8.31 ± 0.28^b^	58.03 ± 0.22^a^	−1.09 ± 0.10^d^	13.90 ± 0.01^c^
Cg	62.99 ± 0.79^ab^	4.82 ± 0.12^c^	45.86 ± 0.21^b^	9.50 ± 0.30^ab^	54.76 ± 0.11^c^	0.15 ± 0.01^b^	16.38 ± 0.13^a^
Cj	62.31 ± 0.59^ab^	5.24 ± 0.02^b^	47.20 ± 0.93^ab^	9.91 ± 0.28^ab^	48.02 ± 0.15^d^	0.32 ± 0.04^b^	14.97 ± 0.06^b^
Cp	64.87 ± 0.73^a^	4.56 ± 0.01^c^	38.70 ± 0.93^c^	12.43 ± 1.35^a^	56.15 ± 0.06^b^	−0.41 ± 0.04^c^	16.75 ± 0.18^a^
Cs	64.18 ± 0.47^ab^	4.73 ± 0.01^c^	45.03 ± 0.21^b^	11.05 ± 0.22^ab^	48.42 ± 0.20^d^	1.00 ± 0.04^a^	11.28 ± 0.07^d^

*Note:* Mean ± SE. Superscripts within the same column that differ indicate statistically significant differences (*p* < 0.05). *n* = 3; each value represents the mean of three replicates.

Abbreviations: Cc, control group with no vinegar; Cg, meats marinated with marinades prepared with grapefruit vinegar; Cj, meats marinated with marinades prepared with jujube vinegar; Cp, meats marinated with marinades prepared with pineapple vinegar; Cs, meats marinated with marinades prepared with strawberry vinegar.

### 3.3 Color Properties of Spent Hen Meat

Several factors, including genetics, age at slaughter, sex, environmental conditions, diet, and pre‐slaughter stress, affect the color of poultry meat (Qamar et al. 2019). The color parameters of the vinegars are presented in Table 1, while the color parameters of sample treated with different vinegar‐based marinades are provided in Table 2. *L* values were higher in the control group (Cc) than in the marinated samples. Given that vinegars are naturally colored, it is not surprising that meat marinated with them appeared darker. The Cj and Cs groups exhibited the lowest L* values, measuring 48.02 and 48.42, respectively. The natural color of grapefruit and strawberry is dark; therefore, it makes sense that both the marinade and the chicken meat took on a deeper color tone after treatment. Kadıoğlu et al. (2019) reported that marinating chicken meat with pineapple juice increased the L* value of cooked meat up to 80 min, after which it significantly declined with extended marination time. It is hypothesized that the transfer of pigments from the vinegar to the surface of the breast meat during marination is influenced by factors such as the vinegar's pH, color, and composition (Fencioğlu et al. 2022).

The a* parameters of the Cc group were considerably lower compared to those of the Cg, Cj, Cp, and Cs groups (*p* < 0.05). Spent hen meats treated with strawberry vinegar‐based marinades displayed the highest a* parameters and the lowest b* parameters. The Cs group showed significantly higher a values compared to the other treatments, likely due to the strong red pigmentation characteristic of strawberry vinegar. In contrast, neither concentration of apple vinegar nor 0.2 M acetic acid significantly affected the a values of chicken breast meat (Unal et al. 2020).

The b* parameters of the spent hen meats treated in various marinades ranged from 11.28 to 16.75. The b* value of the vinegar‐marinated meats was significantly higher (*p* < 0.05) than that of the Cc group, except for the Cs group. The meats treated with grapefruit and pineapple vinegar‐based marinades displayed the highest b* values (*p* < 0.05). This observation suggests that the color characteristics of spent hen meat may be influenced by the pigments present in the fruit vinegar‐based marinades. Nonetheless, the b values did not differ significantly between the Cg and Cp treatments. In a separate study, where fruit juices were used as marinades, the b* values of turkey breast meat ranged from 1.91 to 23.83 (Gök and Bor 2016). These findings confirm that the composition of marination liquids—particularly their color and acidity—plays a critical role in determining the color characteristics of meat, as highlighted by previous studies (Gök and Bor 2016; Unal et al. 2020; Fencioğlu et al. 2022).

While marination—particularly with acidic, fruit‐based ingredients—can affect heme proteins in meat, post‐mortem muscle tissue primarily contains myoglobin, not hemoglobin, as the dominant pigment (Suman and Joseph 2013). Hemoglobin, present only in trace amounts from residual blood, plays a minor role in meat color. Although acidic marinades may cause conformational changes in proteins—such as denaturation or oxidation of heme groups—hemoglobin levels or structural alterations were not specifically measured in this study. Instead, color changes were evaluated instrumentally (L*, a*, b*), which indirectly reflect the state of heme pigments. These changes may be attributed to myoglobin oxidation (e.g., to metmyoglobin), pH shifts, or pigment interactions with fruit‐based phenolic compounds rather than direct conformational effects on hemoglobin.

### 3.4 Moisture Content, Cooking Loss, and Water Holding Capacity

The moisture content, CL, and WHC of the spent hen meats are displayed in Table 2. The moisture content of the spent hen meat varied between 61.04% and 64.87%. The Cp group demonstrated a significantly higher moisture content (*p* < 0.05) than the other samples. Ismail‐Fitry and Afifi Ismail (2018) also noted that beef moisture levels rose with the use of higher concentrations of jujube extract. The increased moisture content in the marinated sample could be attributed to an enhancement in its hydrophilic properties.

Table 2 illustrates that spent hen meats treated with vinegar‐based marinades showed a significant (*p* < 0.05) reduction in CL when compared to the Cc group. CL, a significant concern for the industry, leads to considerable weight loss in carcass meat portions. CL values of the meats ranged between 38.70% and 49.65%. The Cp group exhibited significantly lower cooking loss (*p* < 0.05) compared to all other treatment groups. This effect was likely due to the joint impact of reduced muscle pH and enhanced myosin breakdown. Myosin is crucial for water retention and preserving the integrity of myofibrils, which serve as the primary structure for trapping water within muscle fibers (Liu et al. 2011). Unal et al. (2020) reported that marinating chicken breast with apple cider vinegar reduced CL and improved tenderness, indicating vinegar's effectiveness in enhancing poultry quality.

WHC is a crucial quality parameter in meat processing, impacting fundamental attributes such as color, texture, and tenderness in meat and meat products (Unal et al. 2020). Additionally, WHC plays a significant role in determining the economic value of the product. The Cp group exhibited the highest WHC. Nonetheless, the WHC values showed no notable variation among the Cg, Cj, and Cs groups. According to Unal et al. (2020), the incorporation of acetic acid notably enhanced the WHC of the meat samples. Lower pH levels are known to shift the isoelectric point of meat proteins, which can enhance their ability to retain water. This mechanism may help explain the observed increase in moisture retention (Kahraman et al. 2012).

### 3.5 TPA and Warner–Bratzler Results

The textural properties of meat play a crucial role in determining consumer preference for meat products. The results of the texture profile and Warner–Bratzler values are shown in Table 3. Marination with different types of vinegar significantly (*p* < 0.05) affected the hardness, gumminess, and chewiness properties of the spent hen meats. The hardness of the Cg, Cj, Cp, and Cs groups was significantly lower than that of the Cc group. This decrease can be ascribed to the acidic marination process, which promotes the degradation and solubilization of connective tissues, resulting in a more tender texture due to the effect of vinegar. Pineapple and strawberry vinegars were found to be the most effective in improving the tenderness of the meat samples. Chang et al. (2010) suggested that the enhancement of textural properties through marination could be linked to the presence of organic acids. Similarly, Sengun et al. (2021) reported that the hardness parameters of meat can be influenced by the type of vinegar used in marination, due to the organic acid content of the vinegar samples. Latoch (2020) stated that the improvement in tenderness results from the weakening of the muscle structure. On the other hand, the use of these marinades did not significantly affect springiness and cohesiveness values (*p* > 0.05). The lowest resilience value was recorded in the Cp group. Likewise, both concentrations of apple cider vinegar and 0.2 M acetic acid significantly reduced the hardness of chicken breast meat compared to the control, while no significant differences were found in cohesiveness among the treated groups (Unal et al. 2020).

**TABLE 3:** Texture profile analysis and Warner–Bratzler results of spent chicken meats.

Samples	TPA parameters						Warner–Bratzler parameters	
	Hardness (N)	Springiness (mm)	Cohesiveness	Gumminess (N)	Chewiness (Nxmm)	Resilience	WBSF (N)	WBSE (N.s)
Cc (Control)	76.16 ± 1.67^a^	0.86 ± 0.02^a^	0.80 ± 0.02^a^	61.48 ± 1.59^a^	48.37 ± 1.17^a^	0.39 ± 0.03^a^	30.59 ± 4.16^a^	144.35 ± 9.99^a^
Cg	41.62 ± 1.39^c^	0.82 ± 0.01^a^	0.77 ± 0.01^a^	32.52 ± 1.51^c^	26.77 ± 1.79^c^	0.35 ± 0.01^ab^	15.63 ± 3.01^ab^	73.25 ± 4.12^bc^
Cj	56.58 ± 1.46^b^	0.81 ± 0.02^a^	0.81 ± 0.02^a^	51.48 ± 1.37^b^	39.36 ± 0.64^b^	0.38 ± 0.01^ab^	21.27 ± 3.18^ab^	86.48 ± 4.53^b^
Cp	33.67 ± 1.76^d^	0.80 ± 0.01^a^	0.74 ± 0.03^a^	27.30 ± 1.26^c^	25.66 ± 1.29^c^	0.31 ± 0.01^b^	10.83 ± 1.00^b^	42.76 ± 1.75^c^
Cs	34.30 ± 1.58^d^	0.84 ± 0.02^a^	0.82 ± 0.04^a^	29.75 ± 1.72^c^	27.60 ± 1,00^c^	0.38 ± 0.03^ab^	11.47 ± 2.68^b^	58.32 ± 3.31^bc^

*Note:* Mean ± SE. Superscripts within the same column that differ indicate statistically significant differences (*p* < 0.05). *n* = 3; each value represents the mean of three replicates.

Abbreviations: Cc, control group with no vinegar; Cg, meats marinated with marinades prepared with grapefruit vinegar; Cj, meats marinated with marinades prepared with jujube vinegar; Cp, meats marinated with marinades prepared with pineapple vinegar; Cs, meats marinated with marinades prepared with strawberry vinegar.

The Warner–Bratzler (Table 3) parameters exhibited a decreasing trend in spent hen meats marinated with vinegar‐based marinades. The Cp and Cs groups exhibited the lowest Warner–Bratzler shear force (WBSF) values, measuring 10.83 N and 11.47 N, respectively. Subsequently, the other vinegar treatments followed, with the control group showing the highest WBSF value of 30.59 N. Fruit vinegars decreased the WBSE parameters of the meat samples. The Cp group exhibited the lowest WBSE values (42.76 N.s) in the sample. Unal et al. (2020) reported that marinating chicken breast meat with apple cider vinegar led to a significant reduction in Warner–Bratzler shear force (WBSF) values compared to untreated samples. This improvement in tenderness was associated with the vinegar's acidity, which facilitates collagen breakdown and softens muscle fibers.

### 3.6 Scanning Electron Microscopy Analysis of Meat Microstructure

**FIGURE 1:**
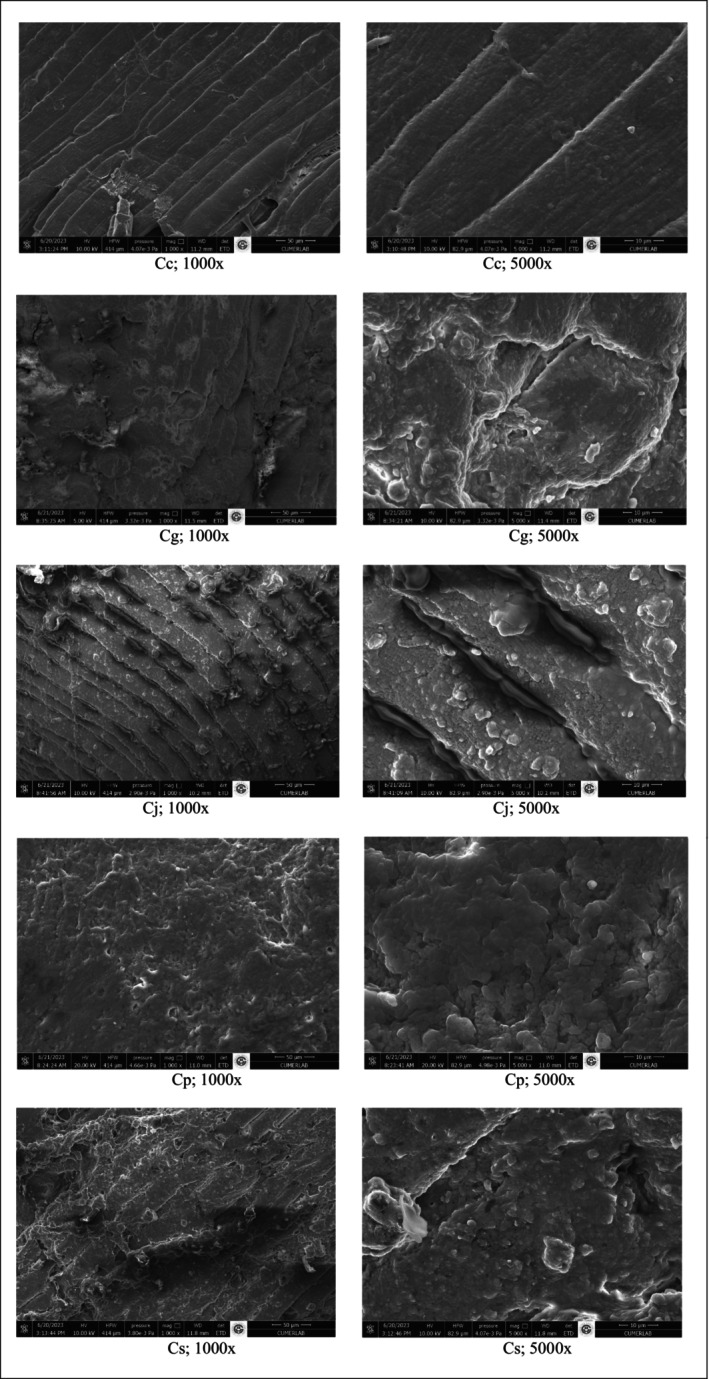
Microstructure characterization of spent chicken meats. Cc, control group with no vinegar; Cg, meats marinated with marinades prepared with grapefruit vinegar; Cj, meats marinated with marinades prepared with jujube vinegar; Cp, meats marinated with marinades prepared with pineapple vinegar; Cs, meats marinated with marinades prepared with strawberry vinegar.

Figure 1 displays scanning electron microscopy images of spent hen meat samples. Proteolytic degradation causes the disruption of myofibrils, contributing to the tenderisation of meat. Therefore, vinegar‐treated meat samples were analyzed using scanning electron microscopy to identify the affected proteins. The fibers in the meats marinated with the marinade (control group), which did not contain vinegar, were tightly bound together. As shown in Figure 1 Cc, the SEM images of the Cc group at ×1000 and ×5000 magnifications reveal that there is no gap between the filaments of the spent hen meat, indicating its hardness. In contrast, the SEM images of spent hen meat marinated with different types of fruit vinegars reveal significant deformation and cavities between the fibers, due to the disruption of connective tissues. The SEM findings revealed that marination with vinegar‐based marinades led to irregular muscle fibers, contributing to increased meat tenderness. The Cp group demonstrated the most pronounced effect of vinegar treatment, followed by the Cs group. Furthermore, its higher moisture content and WHC contributed to a reduced hardness value. Likewise, Sunantha and Saroat (2011) suggested that the observed gaps between muscle fiber bundles could result from the breakdown of the sarcolemma and endomysial collagen. The presence of organic acids in vinegars can affect meat texture throughout the marination process. Chang et al. (2010) noted that treating meat with mild organic acids resulted in a reduction in both muscle fiber diameter and wall thickness, while also causing disorganization of collagen structures. An SEM image similar to the one observed in this study was reported by Mazaheri Kalahrodi et al. (2021) for beefsteak chucks treated with asparagus juice and balsamic vinegar, and by Unal et al. (2023) for cow meat marinated with different types of vinegar.

### 3.7 Microbiological Quality

Table 4 shows the microbiological changes in unmarinated raw meat, marinated raw meat, and marinated cooked meat samples. The initial counts of total TMAB, TPAB, total coliform bacteria, *E. coli* , and yeast and mold in raw spent hen meat were recorded as 3.21, 2.70, 2.96, 1.76, and < 1 log CFU/g, respectively. Marination with fruit vinegars caused a significant reduction in microbial counts. The antibacterial characteristics of vinegars, along with their acetic acid content and pH, have been assessed against a range of bacterial strains (Bakir et al. 2017; Sarker et al. 2021). Moreover, previous research in the literature have investigated the antimicrobial properties of marination treatments using acidic marinades (Turan and Şimşek 2021). Vinegars demonstrated greater effectiveness in reducing microbial counts compared to the Cc group. The antibacterial activity of vinegars may be attributed to their acetic acid content and pH level. The process of microbial inactivation by organic acids, such as acetic acid, is widely recognized. This involves the undissociated organic acid (HA) penetrating the cell membrane, depending on the internal pH, and subsequently dissociating into (H^+^) and (A^−^) ions within the cell. Protons increase the acidity, and the resulting enhancement of cytoplasmic acidity leads to cell damage, as well as the modification or denaturation of enzymes and structural proteins (Bakir et al. 2017).

**TABLE 4:** Microbial counts of unmarinated raw meats, marinated raw and cooked meats (log CFU/g).

Samples		Total mesophilic aerobic bacteria (TMAB)	Total psychotropic aerobic bacteria (TPAB)	Total coliform bacteria	*Escherichia coli*	Yeast and mold
Unmarinated raw meat (initial)		3.21 ± 0.35^a^	2.70 ± 0.11^a^	2.96 ± 0.09^a^	1.76 ± 0.28^a^	< 1
Cc	Marinated raw meat	2.83 ± 0.05^a^	2.44 ± 0.11^a^	2.46 ± 0.19^a^	1.55 ± 0.16^a^	< 1
	Cooked	< 2^b^	< 2^b^	< 1^b^	< 1^b^	< 1
Cg	Marinated raw meat	< 2^b^	< 2^b^	< 1^b^	< 1^b^	< 1
	Cooked	< 2^b^	< 2^b^	< 1^b^	< 1^b^	< 1
Cj	Marinated raw meat	< 2^b^	< 2^b^	< 1^b^	< 1^b^	< 1
	Cooked	< 2^b^	< 2^b^	< 1^b^	< 1^b^	< 1
Cp	Marinated raw meat	< 2^b^	< 2^b^	< 1^b^	< 1^b^	< 1
	Cooked	< 2^b^	< 2^b^	< 1^b^	< 1^b^	< 1
Cs	Marinated raw meat	< 2^b^	< 2^b^	< 1^b^	< 1^b^	< 1
	Cooked	< 2^b^	< 2^b^	< 1^b^	< 1^b^	< 1

*Note:* Mean ± SE. Superscripts within the same column that differ indicate statistically significant differences (*p* < 0.05). *n* = 3; each value represents the mean of three replicates.

Abbreviations: Cc, control group with no vinegar; Cg, meats marinated with marinades prepared with grapefruit vinegar; Cj, meats marinated with marinades prepared with jujube vinegar; Cp, meats marinated with marinades prepared with pineapple vinegar; Cs, meats marinated with marinades prepared with strawberry vinegar.

A significant difference (*p* < 0.05) was observed between the raw and cooked meats, as presented in Table 4. At the end of the cooking process, no notable variations (*p* > 0.05) were found among the different treatment groups. Following cooking, the TMAB and TPAB levels in these samples dropped to below 2 log CFU/g, while the total coliform bacteria and *E. coli* counts were reduced to under 1 log CFU/g. The reduction in bacterial counts may result from the breakdown of the plasma membrane, protein denaturation, and alterations in the permeability of the microbial cell wall (Chong and Cossius 1983). Thermal methods like sous vide cooking effectively reduce microbial loads in poultry. Recent data show reductions of 4.82, 4.34, and 1.70 log CFU/g for *Campylobacter*, *Salmonella*, and *Clostridium* spores, respectively (Romeo et al. 2024). Similarly, hot water spray at 60°C and 450 psi lowered total aerobic bacteria, *Enterobacteriaceae*, and *E. coli* by up to 2.50 log CFU/carcass (Cosby et al. 2024). Effectiveness varies with microbial type and treatment conditions.

### 3.8 Sensory Scores

**FIGURE 2:**
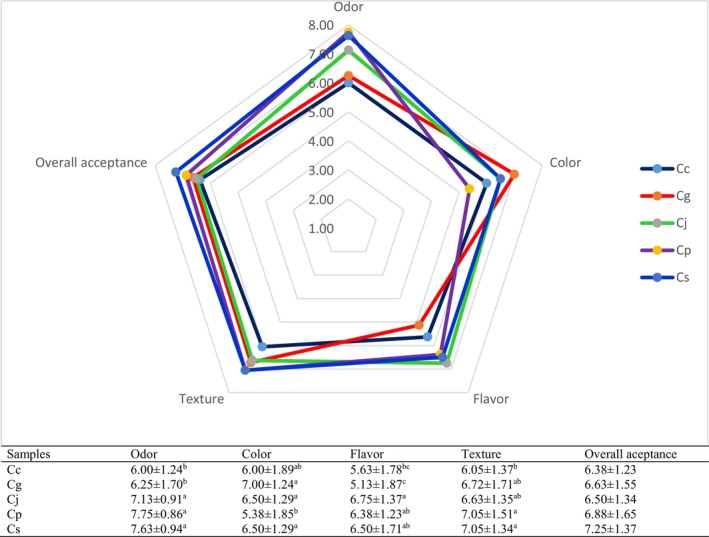
Sensory properties of spent chicken meat. Mean ± SE. Superscripts within the same column that differ indicate statistically significant differences (*p* < 0.05). *n* = 60; each value represents the mean of 60 replicates. Cc, control group with no vinegar; Cg, meats marinated with marinades prepared with grapefruit vinegar; Cj, meats marinated with marinades prepared with jujube vinegar; Cp, meats marinated with marinades prepared with pineapple vinegar; Cs, meats marinated with marinades prepared with strawberry vinegar.

Figure 2 illustrates that the sensory ratings for all spent hen meats varied between 5.13 and 7.75. The application of these marinades affected the odor, color, flavor, and texture values (*p* > 0.05), whereas no significant impact was observed on overall acceptance (*p* < 0.05). Smell plays a vital role in the quality of meat sample, given the strong relationship between spoilage and odor (Sarfraz et al. 2021). The Cg group recorded the lowest flavor scores (*p* < 0.05). This was likely due to the distinctive flavor of grapefruit vinegar competing with the inherent flavor of spent hen meat at cooking temperatures. Lytou et al. (2017) demonstrated that marinades prepared with a variety of vinegars enhance the sensory characteristic of meat products. Spent hen meats marinated with vinegar samples exhibited higher texture scores than those of the Cc group, reflecting improved tenderness and overall eating quality. Spent hen meats marinated with pineapple and strawberry vinegars received the highest texture scores (*p* < 0.05). This result was consistent with the SEM images and TPA parameters of the meats. While earlier studies suggested that vinegar‐based marinades can enhance the sensory attributes of meat products Lytou et al. (2017) and Aktaş and Kaya (2001) emphasized that the acidity level should remain below 1% to avoid undesirable effects on flavor and aroma.

## 4 Conclusion

Meat marinated with grapefruit, pineapple, and strawberry vinegars demonstrated significantly lower cooking loss compared to the control group. Additionally, pineapple vinegar treatment notably improved moisture content and water holding capacity. Color properties were influenced by the use of these marinades. Vinegar marination reduced the hardness, gumminess, chewiness, resilience, WBSF, and WBSE values of spent hen meat, with pineapple vinegar emerging as the most effective treatment, followed by strawberry vinegar. The scanning electron microscopy images indicated that marination with vinegar‐based marinades caused significant deterioration of muscle fibers, resulting in irregular muscle fibers, which in turn made the meat more tender. The use of vinegars in marination led to a significant reduction in microbial counts. In terms of sensory characteristics, marination with vinegars did not adversely affect the odor, color, flavor, or overall acceptance scores of the samples, but it did enhance their texture scores. Enhancing the marketability of spent hen meat, which is frequently discarded due to its lower quality and tough texture, remains a considerable challenge. The findings suggest that vinegars, particularly pineapple and strawberry vinegar, can act as natural tenderizers for aged spent hen meat while also improving other quality attributes, thereby enhancing its overall consumer acceptability.

## Author Contributions

**Nuran Erdem:** conceptualization, data curation, formal analysis, methodology, investigation, writing – original draft, writing – review and editing.

## Ethics Statement

The author has nothing to report.

## Conflicts of Interest

The author declares no conflicts of interest.

## Data Availability

Data will be made available on request.

## References

[fsn370544-bib-0001] Aktaş, N. , and M. Kaya . 2001. “The Influence of Marinating With Weak Organic Acids and Salts on the Intramuscular Connective Tissue and Sensory Properties of Beef.” European Food Research and Technology 213, no. 2: 88–94. 10.1007/s002170100329.

[fsn370544-bib-0002] AOAC . 2000. AOAC International Official Methods of Analysis of AOAC International. 17th ed, 2417–2877. AOAC International.

[fsn370544-bib-0003] AOAC . 2007. Official Analytical Chemists. 18th ed. Association of Official Analytical Chemists, AOAC International.

[fsn370544-bib-0004] Aviagen . 2021. “Parent Stock Ross 308 Parent Stock Performance Objectives.” Last Access April 27, 2021. https://en.aviagen.com/assets/Tech_Center/Ross_PS/Ross308‐ParentStock‐PerformanceObjectives‐2021‐EN.pdf.

[fsn370544-bib-0005] Bakir, S. , D. Devecioglu , S. Kayacan , G. Toydemir , F. Karbancioglu‐Guler , and E. Capanoglu . 2017. “Investigating the Antioxidant and Antimicrobial Activities of Different Vinegars.” European Food Research and Technology 243: 2083–2094. 10.1007/s00217-017-2908-0.

[fsn370544-bib-0006] Bayram, M. , C. Kaya , E. E. Yücel , B. Er , E. Gülmez , and E. Terzioğlu . 2018. “Pirinç sirkesi ve çeşitli ticari sirkelerin bazı kalite özellikleri.” Akademik Gıda 16, no. 3: 293–300. 10.24323/akademik-gida.475357.

[fsn370544-bib-0007] Bayram, Y. , K. Özkan , and O. Sagdıc . 2020. “Bioactivity, Physicochemical and Antimicrobial Properties of Vinegar Made From Persimmon ( *Diospyros kaki* ) Peels.” Sigma Journal of Engineering and Natural Sciences 38, no. 3: 1643–1652.

[fsn370544-bib-0008] Bhat, Z. F. , J. D. Morton , S. L. Mason , and A. E. D. Bekhit . 2018. “Applied and Emerging Methods for Meat Tenderization: A Comparative Perspective.” Comprehensive Reviews in Food Science and Food Safety 17, no. 4: 841–859. 10.1111/1541-4337.12356. 33350109

[fsn370544-bib-0009] Boondaeng, A. , S. Kasemsumran , K. Ngowsuwan , et al. 2022. “Comparison of the Chemical Properties of Pineapple Vinegar and Mixed Pineapple and Dragon Fruit Vinegar.” Fermentation 8, no. 11: 597. 10.3390/fermentation8110597.

[fsn370544-bib-0010] Chang, H. J. , Q. Wang , G. H. Zhou , X. L. Xu , and C. B. Li . 2010. “Influence of Weak Organic Acids and Sodium Chloride Marination on Characteristics of Connective Tissue Collagen and Textural Properties of Beef Semitendinosus Muscle.” Journal of Texture Studies 41, no. 3: 279–301.

[fsn370544-bib-0011] Chong, G. , and A. R. Cossius . 1983. “A Differential Polarized Flurometric Study of the Effects of High Hydrostatic Pressure Upon the Fluidity of Cellular Membranes.” Biochemistry 22: 409–415. 6297548 10.1021/bi00271a026

[fsn370544-bib-0012] Cosby, D. E. , M. D. McIntyre , J. DeVoll , et al. 2024. “Reduction of Bacterial Load on Broiler Carcasses Using Low‐Volume Fluidic Nozzles in Combination With 60°c Water at 450 Psi Pressure.” Poultry 3, no. 1: 15–25. 10.3390/poultry3010002.

[fsn370544-bib-0013] Fencioğlu, H. , E. Oz , S. Turhan , S. C. Proestos , and F. Oz . 2022. “The Effects of the Marination Process With Different Vinegar Varieties on Various Quality Criteria and Heterocyclic Aromatic Amine Formation in Beef Steak.” Food 11: 3251. 10.3390/foods11203251. PMC9602021 37431000

[fsn370544-bib-0014] Gök, V. , and Y. Bor . 2016. “Effect of Marination With Fruit and Vegetable Juice on the Some Quality Characteristics of Turkey Breast Meat.” Brazilian Journal of Poultry Science 18: 481–488.

[fsn370544-bib-0015] Gökalp, H. Y. , M. Kaya , Y. Tülek , and Ö. Zorba . 1999. Et ve Ürünlerinde Kalite Kontrolü ve Laboratuar Uygulama Kılavuzu. Atatürk Üniversitesi Ziraat Fakültesi Yayınları.

[fsn370544-bib-0016] Herrero, A. M. , J. A. Ordóñez , R. De Avila , B. Herranz , L. De La Hoz , and M. I. Cambero . 2007. “Breaking Strength of Dry Fermented Sausages and Their Correlation With Texture Profile Analysis (TPA) and Physico‐Chemical Characteristics.” Meat Science 77, no. 3: 331–338. 10.1016/j.meatsci.2007.03.022. 22061785

[fsn370544-bib-0017] Ismail‐Fitry, M. R. , and M. Afifi Ismail . 2018. “Application of *Ziziphus Jujube* (Red Date), *Camellia sinensis* (Black Tea) and *Aleurites moluccana* (Candle Nut) Marinades as Beef Meat Tenderizer.” International Journal of Engineering & Technology 7, no. 4.14: 307–311.

[fsn370544-bib-0018] Kadıoğlu, P. , M. Karakaya , K. Unal , and A. S. Babaoğlu . 2019. “Technological and Textural Properties of Spent Chicken Breast, Drumstick and Thigh Meats as Affected by Marinating With Pineapple Fruit Juice.” British Poultry Science 60, no. 4: 381–387. 10.1080/00071668.2019.1621990. 31107112

[fsn370544-bib-0019] Kahraman, T. , A. Bayraktaroglu , G. Issa , T. Ertugrul , E. Bingol , and L. Ergun . 2012. “Effects of Temperature Conditioning and Citrus Juice Marinade on Quality and Microstructure of Aged Beef.” Journal of Food, Agriculture & Environment 10, no. 1: 117–122.

[fsn370544-bib-0020] Kang, M. , J. H. Ha , and Y. Lee . 2020. “Physicochemical Properties, Antioxidant Activities and Sensory Characteristics of Commercial Gape Vinegars During Long‐Term Storage.” Food Science and Technology (Campinas) 40, no. 4: 909–916.

[fsn370544-bib-0021] Kim, H. W. , Y. S. Choi , J. H. Choi , et al. 2013. “Tenderization Effect of Soy Sauce on Beef *M. biceps* Femoris.” Food Chemistry 139, no. 1–4: 597–603. 10.1016/j.foodchem.2013.01.050. 23561150

[fsn370544-bib-0022] Kumar, D. , A. Mishra , A. Tarafdar , et al. 2021. “Protease Catalyzed Production of Spent Hen Meat Hydrolysate Powder for Health Food Applications.” Hindawi Journal of Food Quality 2021: 9247998. 10.1155/2021/9247998.

[fsn370544-bib-0023] Latoch, A. 2020. “Effect of Meat Marinating in Kefir, Yoghurt and Buttermilk on the Texture and Color of Pork Steaks Cooked Sous‐Vide.” Annals of Agricultural Science 65, no. 2: 129–136.

[fsn370544-bib-0024] Lee, S.‐H. , and H.‐Y. Kim . 2021. “Comparison of Quality and Sensory Characteristics of Spent Hen and Broiler in South Korea.” Animals 11: 2565. 10.3390/ani11092565. 34573531 PMC8466627

[fsn370544-bib-0025] Li, X. , M. Ha , R. D. Warner , and F. R. Dunshea . 2021. “Meta‐Analysis of the Relationship Between Collagen Characteristics and Meat Tenderness.” Meat Science 182: 108635. 10.1016/j.meatsci.2021.108635. 34839194

[fsn370544-bib-0026] Liu, C. , Y. Xiong , and G. Rentfrow . 2011. “Kiwifruit Protease Extract Injection Reduces Toughness of Pork Loin Muscle Induced by Freezeethaw Abuse.” LWT‐Food Science and Technology 44: 2026–2031.

[fsn370544-bib-0028] Lytou, A. E. , E. Z. Panagou , and G. J. E. Nychas . 2017. “Effect of Different Marinating Conditions on the Evolution of Spoilage Microbiota and Metabolomic Profile of Chicken Breast Fillets.” Food Microbiology 66: 141–149. 28576362 10.1016/j.fm.2017.04.013

[fsn370544-bib-0029] Martinez, H. A. , R. K. Miller , C. Kerth , and B. E. Wasser . 2023. “Prediction of Beef Tenderness and Juiciness Using Consumer and Descriptive Sensory Attributes.” Meat Science 205: 109292. 10.1016/j.meatsci.2023.109292. 37611462

[fsn370544-bib-0030] Mazaheri Kalahrodi, M. , H. Baghaei , B. Emadzadeh , and M. Bolandi . 2021. “The Combined Effect of Asparagus Juice and Balsamic Vinegar on the Tenderness, Physicochemical and Structural Attributes of Beefsteak.” JFST 58, no. 8: 3143–3153. 10.1007/s13197-020-04817-4 PMC8249547 34294976

[fsn370544-bib-0031] Öztürk, H. İ. 2022. “Kardinal Üzümü, Napolyon Kirazı, Mürdüm Eriği, Kivi ve Şeftali meyvelerinden doğal fermantasyonla sirke üretim potansiyeli: fizikokimyasal ve duyusal özellikler.” Akademik Gıda 20, no. 1: 54–62. 10.24323/akademik-gida.1097836.

[fsn370544-bib-0032] Petek, M. , and E. Çavuşoğlu . 2021. “Carcass Characteristics and Physical Meat Quality Properties of Spent Broiler Breeder Hens and Commercial Spent Layer Hens.” Harran Üniversitesi Veteriner Fakültesi Dergisi 10, no. 2: 172–177. 10.31196/huvfd.996375.

[fsn370544-bib-0033] Polizer Rocha, Y. C. , J. M. Lorenzo , J. C. Barros , J. C. Baldin , and M. A. Trindade . 2019. “Effectof Chicken Meat Replacement by Spent Laying Hen Meat on Physicochemical Properties and Sensorial Characteristics of Fresh Sausage.” British Poultry Science 60, no. 2: 139–145. 10.1080/00071668.2019.1568392. 30646752

[fsn370544-bib-0034] Qamar, A. , S. G. Mohyuddin , A. Hamza , K. A. Lartey , and C. O. Shi . 2019. “Physical and Chemical Factors Affecting Chicken Meat Color.” Pakistan Journal of Science 71: 82–88.

[fsn370544-bib-0035] Romeo, M. , M. Lavilla , and F. Amárita . 2024. “Microbial Food Safety of Sous Vide Cooking Processes of Chicken and Eggs.” Food 13, no. 19: 3187. 10.3390/foods13193187. PMC11475771 39410222

[fsn370544-bib-0036] Sarfraz, J. , A. Å. Hansen , J.‐E. Haugen , et al. 2021. “Biodegradable Active Packaging as an Alternative to Conventional Packaging: A Case Study With Chicken Fillets.” Food 10, no. 5: 1126. 10.3390/foods10051126. PMC8161013 34069511

[fsn370544-bib-0037] Sarkar, B. K. , S. Upadhyay , P. Gogoi , S. Choudhury , and D. Deuri . 2020. “Utilization of Spent Hen in Food Industry—A Review.” International Journal of Current Microbiology and Applied Sciences 9, no. 7: 1442–1451.

[fsn370544-bib-0038] Sarker, M. I. A. , M. A. Hashem , M. A. K. Azad , M. S. Ali , and M. M. Rahman . 2021. “Food Grade Vinegar Acts as an Effective Tool for Short‐Term Meat Preservation.” Meat Research 1, no. 1: 5. 10.55002/mr.1.1.5.

[fsn370544-bib-0039] Sengun, I. Y. , G. Y. Turp , S. N. Cicek , T. Avci , B. Ozturk , and G. Kilic . 2021. “Assessment of the Effect of Marination With Organic Fruit Vinegars on Safety and Quality of Beef.” International Journal of Food Microbiology 336: 108904. 10.1016/j.ijfoodmicro.2020.108904. 33129004

[fsn370544-bib-0040] Somek, O. 2018. “Determination of Effect of Wines Produced From Local Black Grape Varieties on the Shelf Life and *Salmonella typhimurium* in Chicken Meat Marination and Determination of Resveratrol, Quartet and Catechin Levels.” Master Thesis, Ege University, Izmir, Turkey.

[fsn370544-bib-0041] Suman, S. P. , and P. Joseph . 2013. “Myoglobin Chemistry and Meat Color.” Annual Review of Food Science and Technology 4: 79–99. 10.1146/annurev-food-030212-182623. 23190143

[fsn370544-bib-0042] Sunantha, K. , and R. Saroat . 2011. “Application of Bromelain Extract for Muscle Foods Tenderization.” Food and Nutrition Sciences 2, no. 5: 393–401.

[fsn370544-bib-0043] Turan, E. , and A. Şimşek . 2021. “Effects of Lyophilized Black Mulberry Water Extract on Lipid Oxidation, Metmyoglobin Formation, Color Stability, Microbial Quality and Sensory Properties of Beef Patties Stored Under Aerobic and Vacuum Packaging Conditions.” Meat Science 178: 108522. 10.1016/j.meatsci.2021.108522. 33957374

[fsn370544-bib-0044] Unal, K. , E. Alagöz , A. Cabi , and C. Sarıçoban . 2020. “Determination of the Effect of Some Acidic Solutions on the Tenderness and Quality Properties of Chicken Breast Meat.” Selcuk Journal of Agriculture and Food Sciences 34, no. 1: 19–23. 10.15316/SJAFS.2020.190.

[fsn370544-bib-0045] Unal, K. , A. S. Babaoglu , and M. Karakaya . 2023. “Improving the Textural and Microstructural Quality of Cow Meat by Black Chokeberry, Grape, and Hawthorn Vinegar‐Based Marination.” Food Science & Nutrition 11: 6260–6270. 10.1002/fsn3.3566. 37823113 PMC10563726

[fsn370544-bib-0046] Wardlaw, F. B. , G. C. Skelley , M. G. Johnson , and J. C. Acton . 1973. “Changes in Meat Components During Fermentation, Heat Procesing and Drying of a Summer Sausage.” Journal of Food Science 38: 1128–1231.

[fsn370544-bib-0047] Young, L. , and R. Buhr . 2000. “Effect of Electrical Stimulation and Polyphosphate Marination on Drip From Early‐Harvested, Individually Quick‐Frozen Chicken Breast Fillets.” Poultry Science 79, no. 6: 925–927. 10.1093/ps/79.6.925. 10875778

